# Supplementary material for: Negative Regulation of Autophagy during Macrophage Infection by Mycobacterium bovis BCG via Protein Kinase C Activation
Source: Int J Mol Sci. 2024 Mar 9;25(6):3145. doi: 10.3390/ijms25063145 (PMC10970242; doi:10.3390/ijms25063145)
Supplement: Supplementary file 1 [file ijms-25-03145-s001.zip › ijms-2671575-supplementary.pdf]

Supplementary Materials:

Figure S1

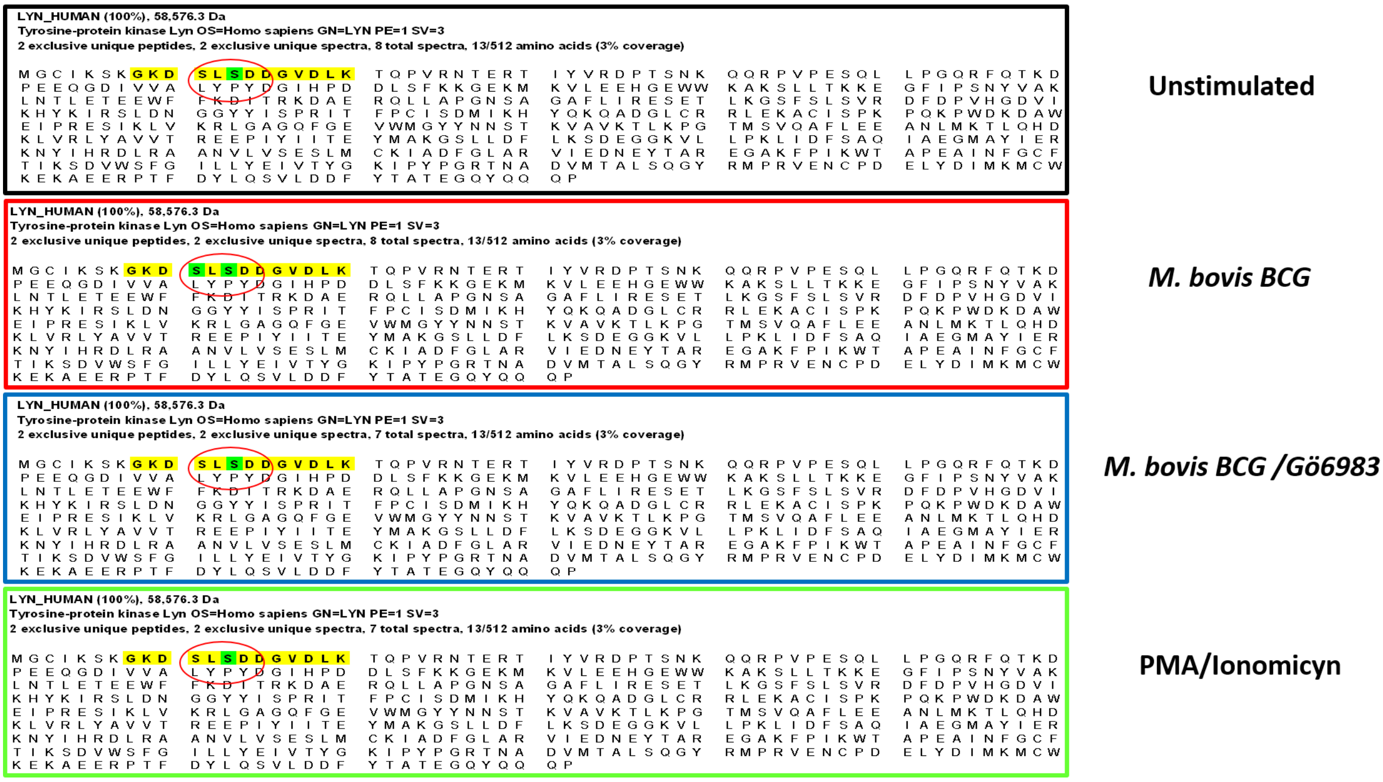

Figure S1: Comparative Analysis of Lyn Protein Sequence. The Lyn protein sequences are depicted under four different treatment conditions as provided by the Scaffold Viewer software. The protein sequence in stimulated cells is highlighted in the black box, while the sequence in cells infected with *M. bovis BCG* is shown in the red box. The blue box represents the sequence in cells previously treated with a PKC inhibitor, and the green box displays the protein sequence in cells treated with PMA/Ionomycin. Phosphorylated peptides identified in mass spectrometry are labeled in yellow, and phosphorylation sites are indicated in green, with 'S' and 'T' denoting serine and threonine residues, respectively.

Figure S2

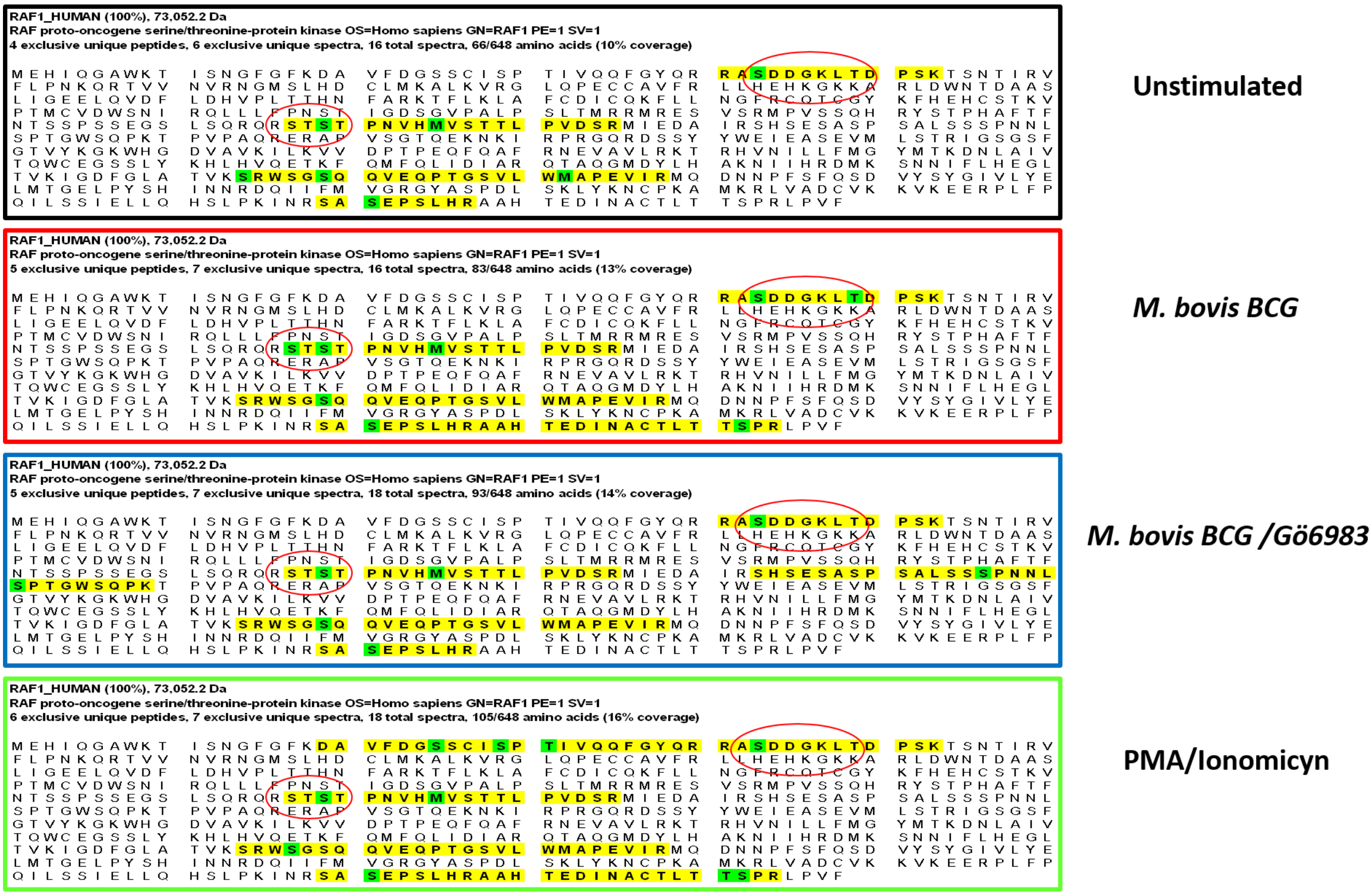

Figure S2: Comparative Analysis of Raf-1 Protein Sequence. The Raf-1 protein sequences are displayed under four distinct treatment conditions as provided by the Scaffold Viewer software. The protein sequence in stimulated cells is highlighted in the black box, while the sequence in cells infected with *M. bovis BCG* is depicted in the red box. The blue box represents the sequence in cells previously treated with a PKC inhibitor, and the green box displays the protein sequence in cells treated with PMA/Ionomycin. Phosphorylated peptides identified in mass spectrometry are denoted in yellow, and phosphorylation sites are indicated in green, with 'S' and 'T' signifying serine and threonine residues, respectively.

.

Figure S3

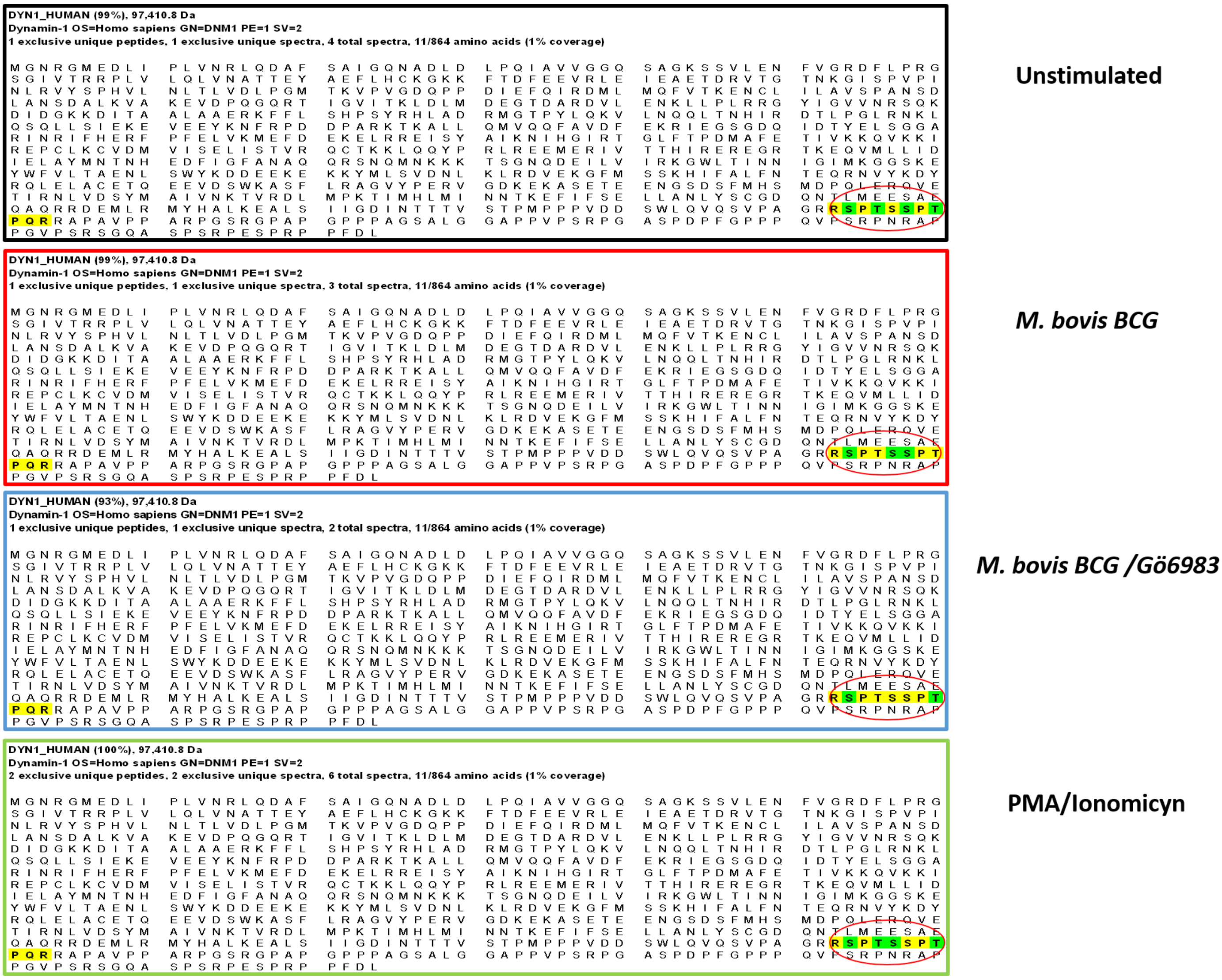

Figure S3: Comparative Analysis of Dynamin-1 Protein Sequence. The Dynamin-1 protein sequences are displayed under four distinct treatment conditions as provided by the Scaffold Viewer software. The protein sequence in stimulated cells is highlighted in the black box, while the sequence in cells infected with *M. bovis BCG* is depicted in the red box. The blue box represents the sequence in cells previously treated with a PKC inhibitor, and the green box displays the protein sequence in cells treated with PMA/Ionomycin. Phosphorylated peptides identified in mass spectrometry are denoted in yellow, and phosphorylation sites are indicated in green, with 'S' and 'T' signifying serine and threonine residues, respectively.

Figure S4

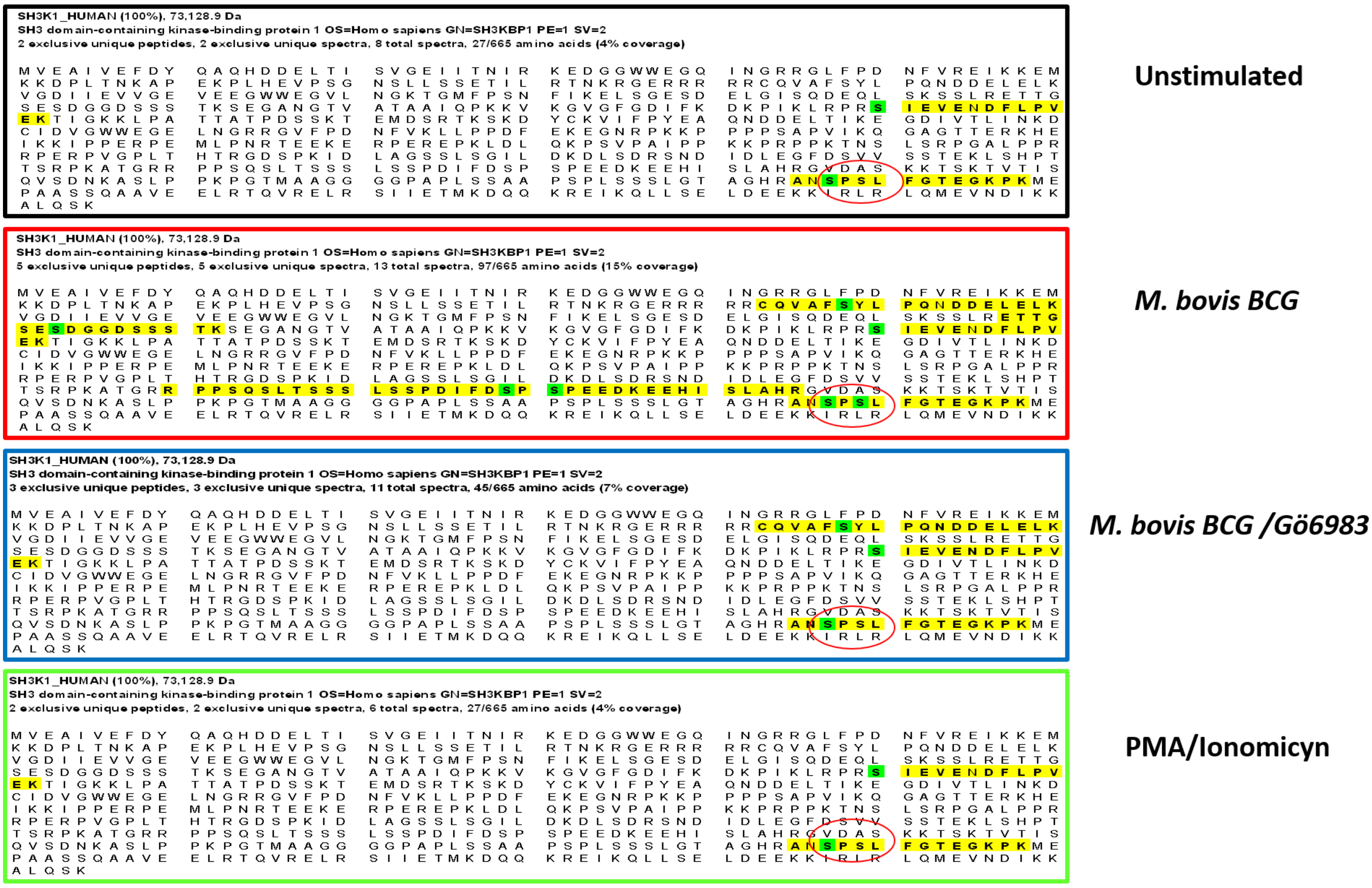

Figure S4: Comparative Analysis of SH3K1 Protein Sequence. The sequence of the SH3K1 protein is presented under four distinct treatment conditions, as generated by the Scaffold Viewer software. The protein sequence observed in stimulated cells is enclosed within the black box, while the red box represents the sequence in cells infected with *M. bovis BCG*. Within the blue box is the sequence in cells previously treated with a PKC inhibitor, and the green box displays the protein sequence in cells treated with PMA/Ionomycin. Phosphorylated peptides, as identified by mass spectrometry, are highlighted in yellow, and phosphorylation events are denoted in green, with 'S' and 'T' indicating serine and threonine residues, respectively.

Figure S5

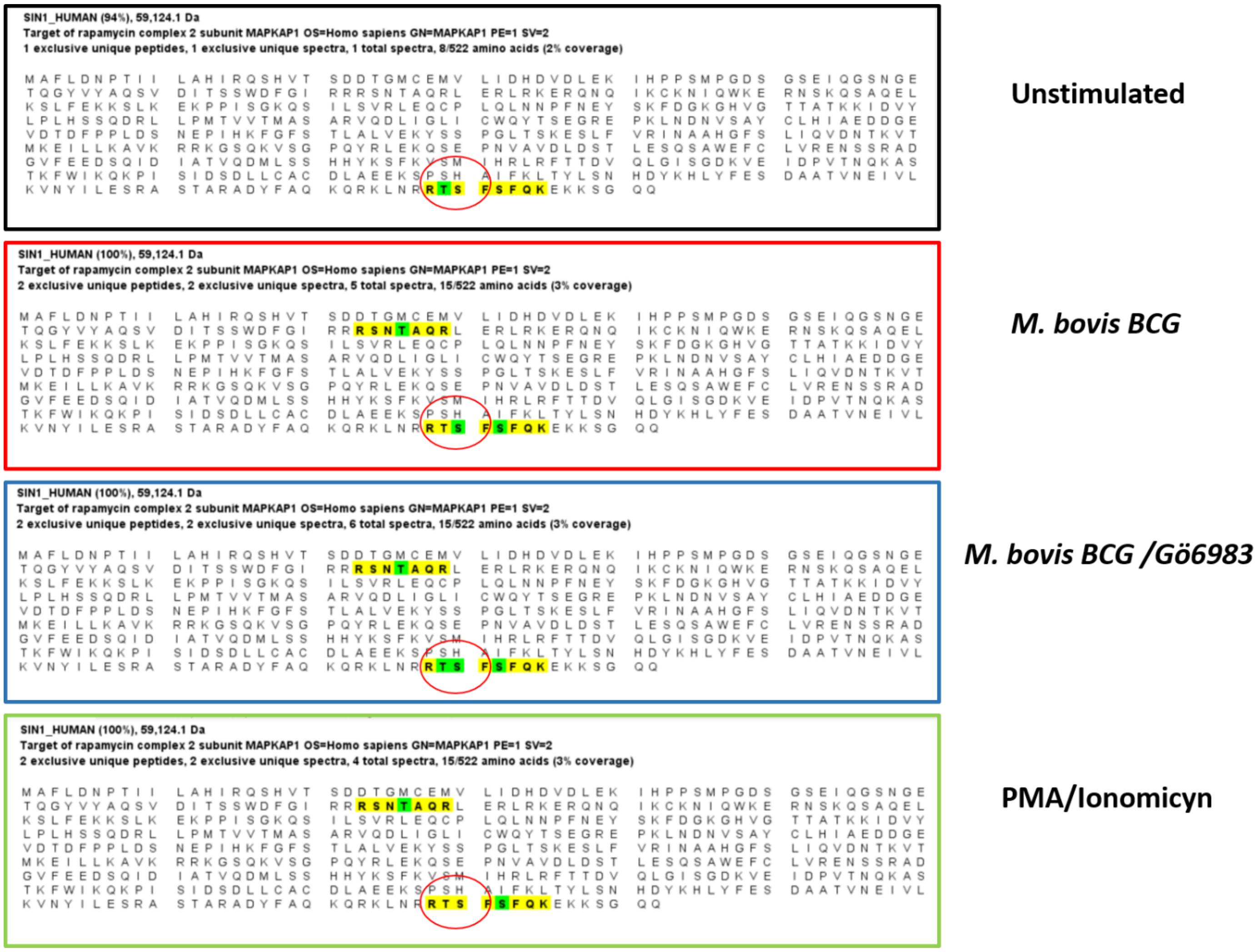

Figure S5: Comparative Analysis of Sin-1 Protein Sequence. The sequence of the Sin-1 protein is presented under four distinct treatment conditions, as generated by the Scaffold Viewer software. The protein sequence observed in stimulated cells is enclosed within the black box, while the red box represents the sequence in cells infected with *M. bovis BCG*. Within the blue box is the sequence in cells previously treated with a PKC inhibitor, and the green box displays the protein sequence in cells treated with PMA/Ionomycin. Phosphorylated peptides, as identified by mass spectrometry, are highlighted in yellow, and phosphorylation events are denoted in green, with 'S' and 'T' indicating serine and threonine residues, respectively.

Figure S6

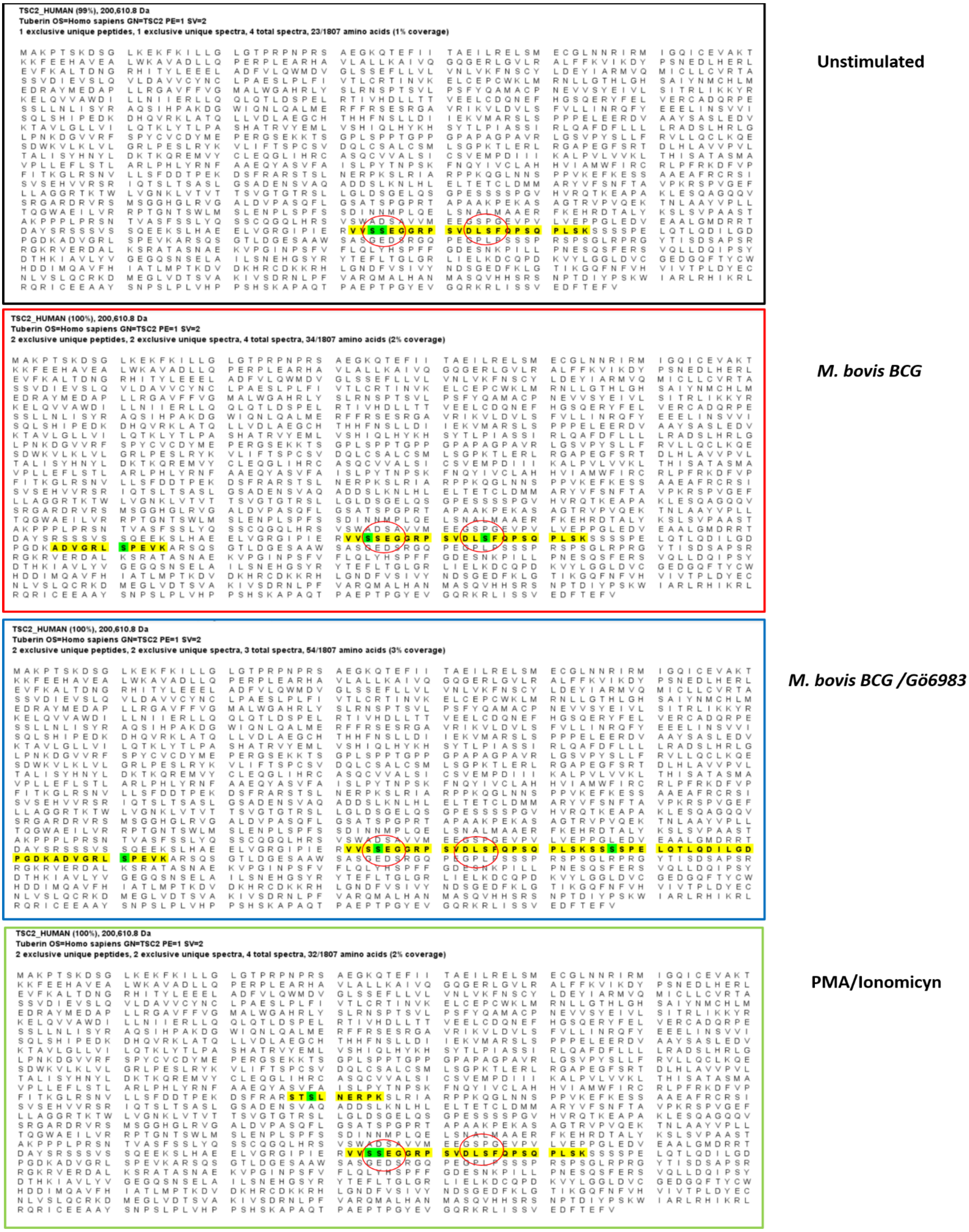

TSC2_HUMAN (99%), 200,610.8 Da
Tuberin OS=Homo sapiens GN=TSC2 PE=1 SV=2
1 exclusive unique peptides, 1 exclusive unique spectra, 4 total spectra, 23/1807 amino acids (1% coverage)
Unstimulated
TSC2_HUMAN (100%), 200,610.8 Da
Tuberin OS=Homo sapiens GN=TSC2 PE=1 SV=2
2 exclusive unique peptides, 2 exclusive unique spectra, 4 total spectra, 34/1807 amino acids (2% coverage)
M. bovis BCG
TSC2_HUMAN (100%), 200,610.8 Da
Tuberin OS=Homo sapiens GN=TSC2 PE=1 SV=2
2 exclusive unique peptides, 2 exclusive unique spectra, 3 total spectra, 54/1807 amino acids (3% coverage)
M. bovis BCG /Gö6983
TSC2_HUMAN (100%), 200,610.8 Da
Tuberin OS=Homo sapiens GN=TSC2 PE=1 SV=2
2 exclusive unique peptides, 2 exclusive unique spectra, 4 total spectra, 32/1807 amino acids (2% coverage)
PMA/Ionomicyn

Figure S6: Comparative Analysis of Tsc2 Protein Sequence. This figure illustrates the Tsc2 protein sequence under four distinct treatment conditions, which have been generated using the Scaffold Viewer software. The protein sequence from stimulated cells is enclosed within the black box, while the red box represents the sequence in cells infected with *M. bovis BCG*. Within the blue box, you can see the sequence from cells that were previously treated with a PKC inhibitor, and in the green box, you'll find the protein sequence from cells treated with PMA/Ionomycin. Notably, phosphorylated peptides identified through mass spectrometry are highlighted in yellow, and the specific phosphorylation events are indicated in green, with 'S' and 'T' denoting serine and threonine residues, respectively.

Figure S7

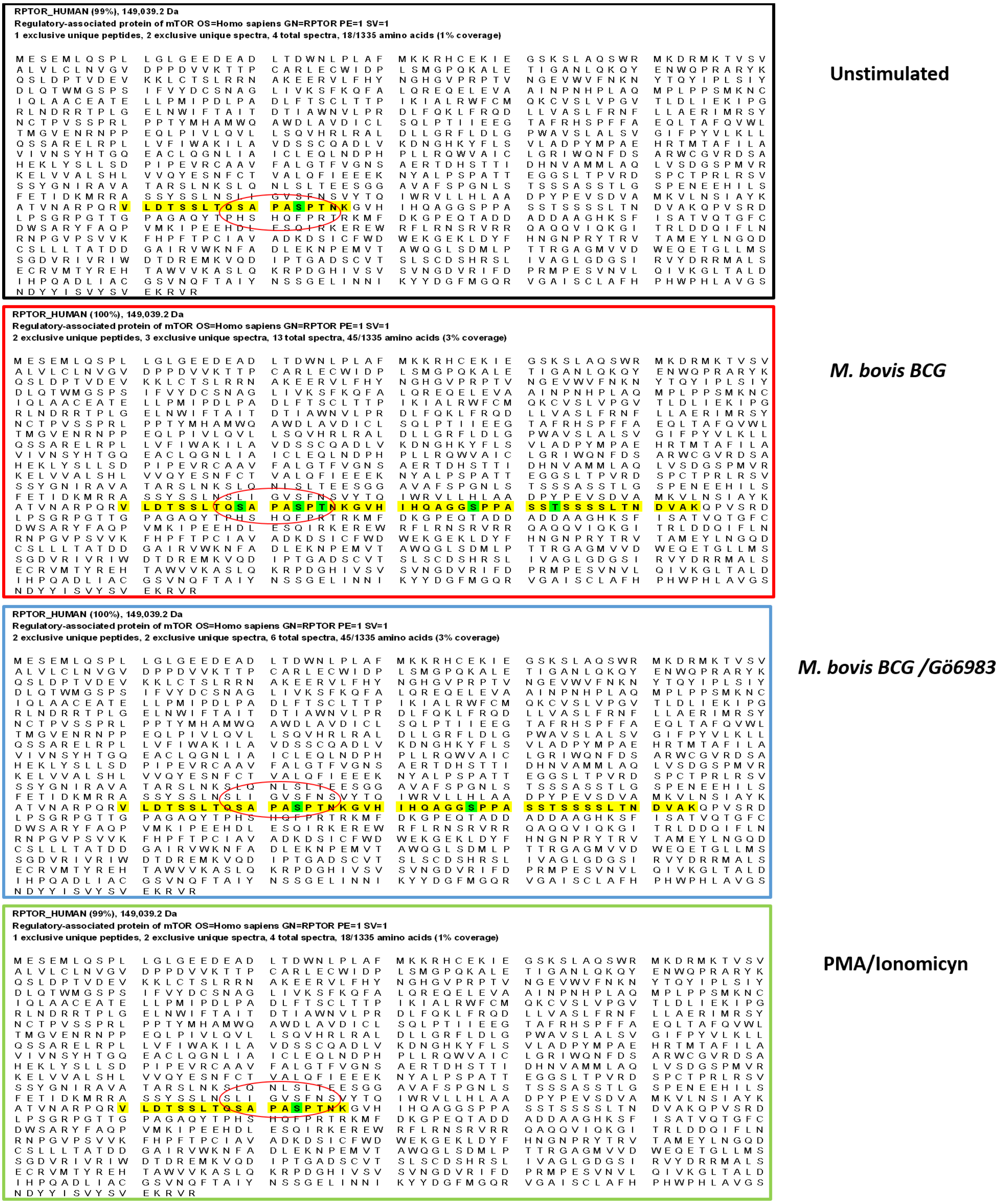
RPTOR_HUMAN (99%), 149,039.2 Da
Regulatory-associated protein of mTOR OS=Homo sapiens GN=RPTOR PE=1 SV=1
1 exclusive unique peptides, 2 exclusive unique spectra, 4 total spectra, 18/1335 amino acids (1% coverage)
Unstimulated
RPTOR_HUMAN (100%), 149,039.2 Da
Regulatory-associated protein of mTOR OS=Homo sapiens GN=RPTOR PE=1 SV=1
2 exclusive unique peptides, 3 exclusive unique spectra, 13 total spectra, 45/1335 amino acids (3% coverage)
M. bovis BCG
RPTOR_HUMAN (100%), 149,039.2 Da
Regulatory-associated protein of mTOR OS=Homo sapiens GN=RPTOR PE=1 SV=1
2 exclusive unique peptides, 2 exclusive unique spectra, 6 total spectra, 45/1335 amino acids (3% coverage)
M. bovis BCG /Gö6983
RPTOR_HUMAN (99%), 149,039.2 Da
Regulatory-associated protein of mTOR OS=Homo sapiens GN=RPTOR PE=1 SV=1
1 exclusive unique peptides, 2 exclusive unique spectra, 4 total spectra, 18/1335 amino acids (1% coverage)
PMA/Ionomicyn

Figure S7: Comparative Analysis of Raptor Protein Sequence. This figure presents the protein sequence of Raptor under four distinct treatment conditions, which have been visualized using the Scaffold Viewer software. Within the black box, you can observe the protein sequence from stimulated cells, while the red box corresponds to the sequence in cells infected with *M. bovis BCG*. The blue box encompasses the sequence in cells previously treated with a PKC inhibitor, and the green box represents the protein sequence in cells treated with PMA/Ionomycin. Notably, phosphorylated peptides identified through mass spectrometry are highlighted in yellow, and specific phosphorylation events are indicated in green, with 'S' denoting serine and 'T' representing threonine residues.

Figure S8

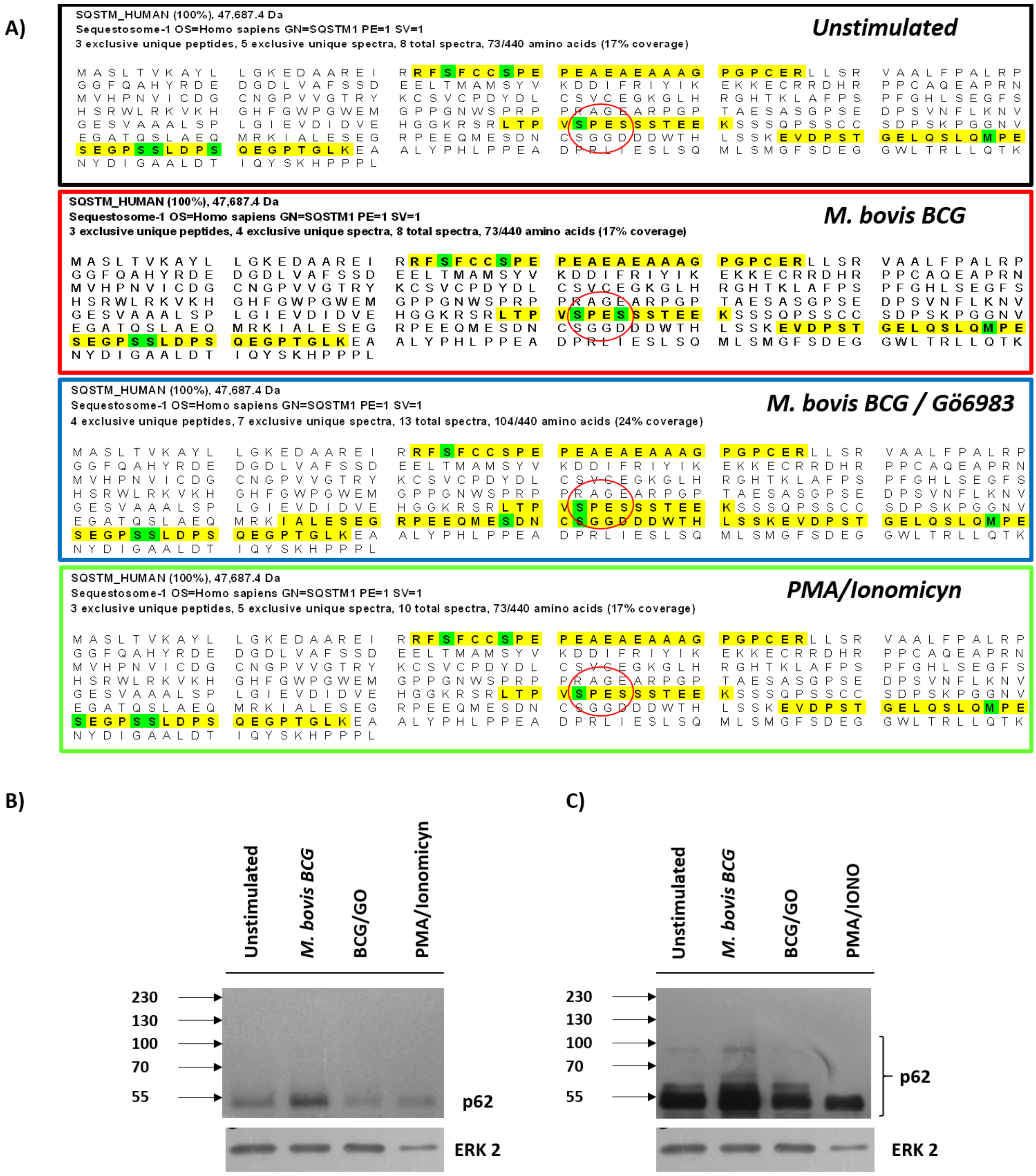

Figure S8: Phosphorylation of Sequestosome-1/p62 by PKC and Its Potential Regulation of Autophagy. Panel A) This panel figure provides insight into the Sequestosome-1/p62 protein sequence across four different treatment conditions, as visualized using the Scaffold Viewer software. The black box displays the protein sequence from stimulated cells, while the red box corresponds to cells infected with *M. bovis BCG*. The blue box encompasses the sequence from cells previously treated with a PKC inhibitor, and the green box represents the protein sequence from cells treated with PMA/Ionomycin. Notably, phosphorylated peptides identified through mass spectrometry are highlighted in yellow, with specific phosphorylation sites labeled in green ('S' for serine and 'T' for threonine residues). **Panel B-C)** To assess the presence of Sequestosome-1/p62 in response to mycobacterial infection, a Western blot assay was performed utilizing a specific antibody. **Panel B)** Displays p62 accumulation upon lower film exposure, whereas **Panel C)** shows p/62 oligomerization upon film overexposure. This analysis sheds light on the phosphorylation of Sequestosome-1/p62 by PKC and its potential role in regulating autophagy.

Figure S9

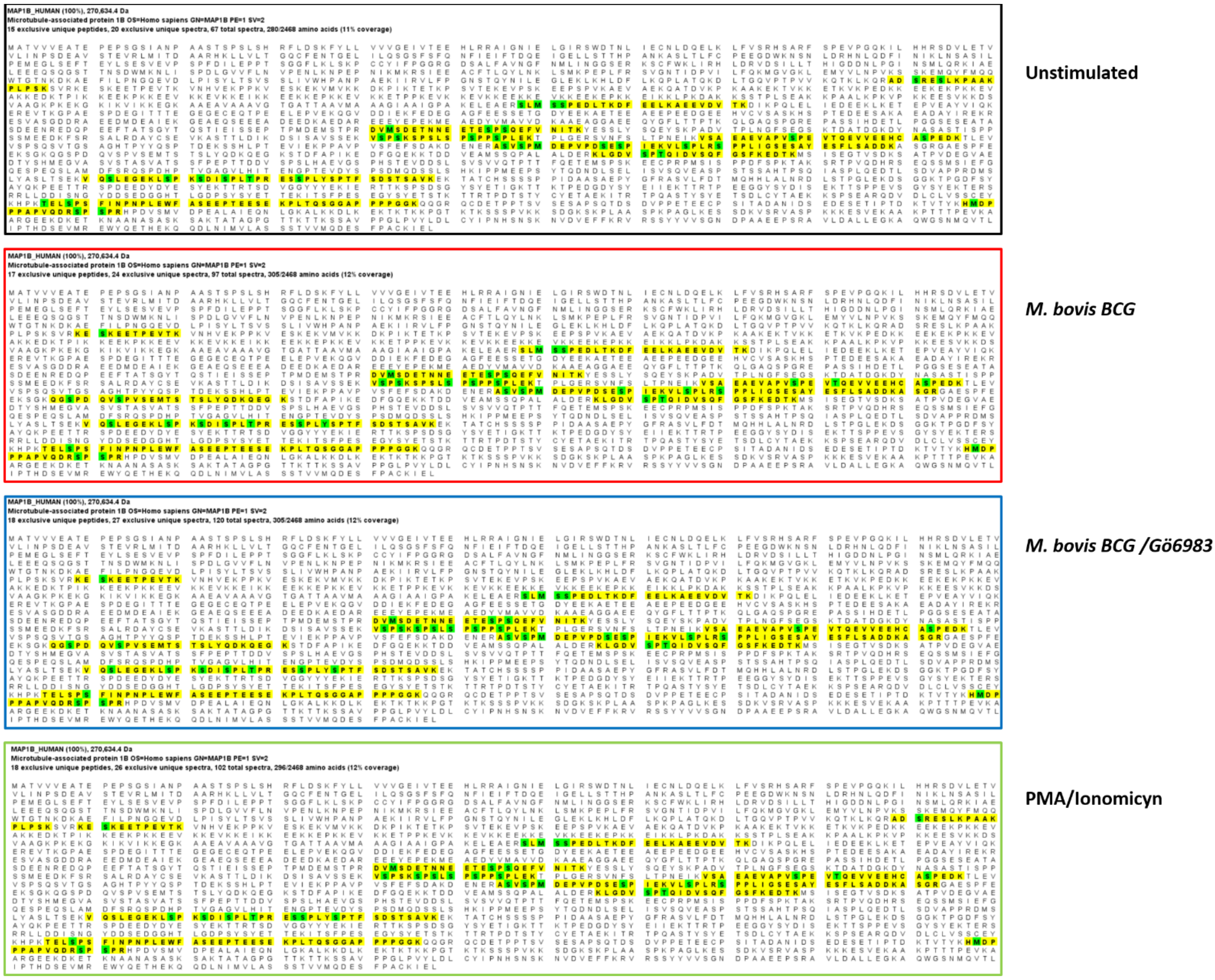

Figure S9: Comparative Analysis of Map-1b Protein Sequence. This figure provides a comparative analysis of the Map-1b protein sequence under four distinct treatment conditions, as visualized using the Scaffold Viewer software. The black box represents the protein sequence observed in stimulated cells, while the red box illustrates the sequence in cells infected with *M. bovis* BCG. The blue box depicts the sequence from cells previously treated with a PKC inhibitor, and the green box showcases the protein sequence in cells treated with PMA/Ionomycin. Notably, phosphorylated peptides identified through mass spectrometry are highlighted in yellow, with specific phosphorylation sites labeled in green ('S' for serine and 'T' for threonine residues).

Table S1

Protein	Gene	ID
C2 domain-containing protein 2- like	C2C2L	O14523
Small glutamine- rich tetratricopepide repeat- containing protein alfa	SGTA	O43765
trans- Golgi network integral membrane protein	TGON2	O43493
Dynamin 2	DYN2	P50570
Osteopontin	OSTP	P10451
Death- associated protein 1	DAP1	P51397
SH3 domain-containing kinase-binding protein	SH3K1	Q96B97
Girdin	GRDN	Q3V6T2
Dynamin 1	DYN1	Q05193
Golgi-specific brefeldin A- Resistance guanine nucleotide exchange factor1	GBF1	Q92538
TBC9 Domain family member 9B	TBC9B	Q66K14
Sequestosome-1	SQSTM	Q13501
Syntaxin- 4	STX4	Q12846
Calcium homeostasis endoplasmic reticulum protein	CHERP	Q8IWX8
Tuberin	TSC2	P49815
Target of Myb protein 1	TOM1	O60784
Gtpase- activating protein and VPS9 domain containing protein	GAPD1	Q14C86
Dedicator of citokinesis protein 8	DOCK8	Q8NF50
Poly (rC)- binding protein 2	PCB P2	Q15366
A-kinase anchor protein 13	AKP13	Q12802
Bcl2- associated transcription factor 1	BCLF1	Q9NYF8
Phosphatidylinositol 3,4,5- trisphosphate 5- phosphatase 1	SHIP1	Q92835
Prelamina- A/C	LMNA	P02545
ProteinNDRG1	NDRG1	Q92597
C-Jun - amino terminal kinase-interacting protein	JIP4	O60271
Stromal membrane- asociated protein 2	SMAP2	Q8WU79
Microtubule associated protein 1B	MAP1B	P46821
Bridging interactor 2	BIN2	Q9UBW5
TBC1 domain family member 10 B	TB10B	Q4KMP7
Phosohatidylinositol 4- kinase type2- alpha	P4K2A	Q9BTU6
Glucocorticoid receptor	GCR	P04150
Niban like protein 1	NIBL1	Q96TA1
Wd repeat- containing protein 44	WDR44	Q5JSH3
Mitogen- activated protein kinase kinase kinase kinase 4	M4K4	O95819
Plastin- 2	PLSL	P13796
RAF proto-oncogene serine/threonine- protein kinase RAF1	RAF1	P04049
Tyrosine-protein kinase Lyn	LYN	P07948
Regulatory- associated protein of mTOR	RPTOR	Q8N122
Protein- methionine sulfoxide oxidase MICAL 1	MICA1	Q8TDZ2
TNF AIP3	TNIP1	Q15025
Was/Wasl- interacting protein family member 2	WIPF2	Q8TF74
Brefeldin A-- inhibited guanine nucleotides-exchange protein	BIG2	Q9Y6D5
FK506-binding protein 15	FKB15	Q5T1M5
Amyloid beta A4 precursor protein-binding family member 1 interacting protein	AB1IP	Q7Z5R6

HLA class I histocompatibility antigen, A-2 alpha chain	HLA	P01892
Eukaryotic translation initiation factor 4E binding protein	4EBP1	Q13541
Zyxin	ZYX	Q15942
Serine threonine- protein Kinase Braf	BRAF	P15056
Eukaryotic translation initiation factor 4 gamma 2	IF4G2	P78344
Target of rapamycin complex 2 subunit MAPKAP1	SIN 1	Q9BPZ7
Protein EVI2B	EVI2B	P34910
Eukaryotic translation initiation factor 4E binding protein 2	4EBP2	Q13542
WD repeat- and domain- containing protein 4	WDFY4	Q6ZS81
WD repeat-containing protein 26	WDR26	Q9H7D7
Insulin receptor sustrate 2	IRS2	Q9Y4H2

Table S1. Proteins involved in biological processes such as endocytosis, vesicular trafficking, autophagy and programmed cell death.
